# Herb Sanqi-Derived Compound K Alleviates Oxidative Stress in Cultured Human Melanocytes and Improves Oxidative-Stress-Related Leukoderma in Guinea Pigs

**DOI:** 10.3390/cells10082057

**Published:** 2021-08-11

**Authors:** Suwei Tang, Lingli Yang, Yasutaka Kuroda, Sylvia Lai, Shaoqiong Xie, Huimin Zhang, Ichiro Katayama

**Affiliations:** 1 Department of Pigmentation Research and Therapeutics, Graduate School of Medicine, Osaka City University, Osaka 5450051, Japan; kellyshadow_tsw@hotmail.com (S.T.); kuroda.yasutaka@kao.com (Y.K.); s-lai@l.wdb-eu.com (S.L.); espaikoffice@gmail.com (I.K.); 2 Department of Dermatology, Shanghai Skin Disease Hospital, Tongji University School of Medicine, Shanghai 200443, China; 18017336630@189.cn; 3 Biological Science Laboratories, Kao Corporation, Kanagawa 2500002, Japan; 4 Department of Dermatology, Shuguang Hospital Affiliated to Shanghai University of Traditional Chinese Medicine, Shanghai 200021, China; zhanghm@shutcm.edu.cn

**Keywords:** Sanqi, melanocytes, oxidative stress, leukoderma

## Abstract

Sanqi, a traditional Chinese herb, is widely used for cardiovascular diseases, and its neuroprotective effects against oxidative stress were recently discovered. The purpose of this study was to investigate whether Sanqi-derived compound K (Sanqi-CK), an active metabolite of Sanqi, could protect melanocytes from oxidative stress. Cultured human primary skin epidermal melanocytes (HEMn-MPs) were treated with hydrogen peroxide (H_2_O_2_) in the presence or absence of Sanqi-CK. Sanqi-CK exhibited protective effects against H_2_O_2_-induced cell death by reducing oxidative stress. In addition, treatment with Sanqi-CK reversed the decreased glutathione reductase activity and decreased ratio of reduced glutathione (GSH)/oxidized glutathione (GSSG) seen in H_2_O_2_-treated melanocytes. Furthermore, topical application of Sanqi-CK alleviated leukoderma in guinea pigs, a disorder characterized by melanocyte cell death resulting from rhododendrol-induced oxidative stress. Taken together, these data suggest that Sanqi-CK protects melanocytes against oxidative stress, and its protective effects are associated with modulating the redox balance between GSH and GSSG and activating glutathione reductase. Thus, Sanqi-CK may be a good candidate for preventing melanocyte loss in oxidative-stress-associated pigmentary disorders.

## 1. Introduction

Oxidative stress is characterized by an imbalance between free radical formation and the body’s antioxidant capacity. Oxidative stress is widely believed to cause serious cellular damage and lead to various pathophysiologic conditions. Skin is the largest barrier organ of our body. It plays an important role in protecting against ultraviolet (UV) irradiation, air pollution, chemicals, pathogens, and other harmful factors that induce oxidative stress. Skin epidermal melanocytes are derived from the spinal portion of the neural crest [1] and are especially vulnerable to oxidative stress, owing to the pro-oxidant state generated during melanin synthesis [2].

Loss of functional melanocytes is the pathologic hallmark of depigmented macules in patients with vitiligo. Vitiligo is the most commonly acquired skin pigmentary disorder, affecting 0.5–1% of the world’s population. It is characterized by white patches of skin resulting from loss of functional epidermal melanocytes [3,4]. The cause of this loss is not fully understood, but both intrinsic (genetic, immunologic, neural, and biochemical) and extrinsic (environmental) factors contribute to vitiligo [4,5]. An increasing body of evidence has suggested that oxidative stress is a major culprit in melanocyte loss; it is considered a key factor in both the onset and progression of vitiligo [6,7,8,9]. High levels of oxidative stress and low levels of enzymatic and nonenzymatic antioxidants have been found in the skin and blood of patients with vitiligo [6,7,8,9,10]. An imbalance between oxidants and antioxidants is suggested as the initial pathogenic event in melanocyte degeneration, playing a crucial role in vitiligo pathogenesis [7].

Accordingly, oxidative stress has been discussed as a promising target for vitiligo treatment. Approaches and treatments using antioxidants to suppress or reverse oxidative stress in the epidermis and even achieve re-pigmentation are being studied. The majority of known exogenous antioxidants are phytochemicals derived from plants. Regulating oxidative stress through administration of naturally occurring substances with antioxidant properties is therefore a potentially promising treatment strategy for vitiligo.

Sanqi is a traditional Chinese medicine derived from the roots of *Panax notoginseng*. It has a long history of medical use spanning over four centuries, and has been referred to as “more precious than gold” [11]. It has been widely used to treat coronary artery disease [12]. Recently, compounds in Sanqi were reported to help reduce beta-amyloid toxicity, slow memory decline, and improve memory and spatial learning in a mouse model of Alzheimer’s disease [13]. In another recent study, Sanqi was shown to have prominent anti-anxiety, antidepressant, and anti-insomnia effects [14]. Sanqi is also considered a promising product for fighting the effects of aging and neurodegenerative diseases [15]. Although vitiligo has features in common with neurodegenerative diseases [1,16], and melanocytes are derived from neural crest cells, no study has heretofore examined the effects of Sanqi in vitiligo.

Sanqi has a distinct ginsenoside profile when compared with other ginseng herbs, including Korean, Siberian, and American ginseng. There are numerous studies about Korean ginseng, whereas reports about Chinese Sanqi are relatively few. The most effective compounds found in Sanqi are four unique saponins, including ginsenoside Rg1, ginsenoside Rb1, ginsenoside Re, and notoginsenoside R1 [11]. As a saponin-rich plant, Sanqi could be a good therapeutic candidate with antioxidant properties. Like many other herbal medicines, Sanqi is usually taken orally. After digestion, its constituents are exposed to gut microflora in the gastrointestinal tract and biotransformed into active metabolites. Compound K (Sanq-CK) is a major bioactive metabolite absorbed into the systemic circulation [17], and possesses much stronger biological activity.

Recent reports indicate that Sanqi-CK has good activity in various oxidative-stress-related conditions. For instance, Sanqi-CK has been shown to alleviate hypolipidemia-associated oxidative stress in high-fat-fed rats through enhancing the activity of hepatic antioxidants via increasing superoxide dismutase levels and glutathione peroxidase activities [18]. Furthermore, Sanqi-CK has demonstrated protective effects against nephropathy in high-fat-fed/streptozotocin-induced mice, acting by significantly reducing oxidative stress and reducing reactive oxygen species (ROS)-mediated activation of inflammasome assembly [19]. Sanqi-CK has also been shown to enhance memory function and reduce neuronal apoptosis by activating the antioxidant system [20]. However, it is unknown whether Sanqi-CK can protect melanocytes from oxidative stress.

Therefore, the current study was conducted to evaluate whether Sanqi-CK protects human melanocytes from oxidative stress and to clarify the underlying molecular mechanisms. We used hydrogen peroxide (H_2_O_2_) to stimulate oxidative stress in normal human primary epidermal melanocytes as an in vitro model and topical rhododendrol to induce oxidative stress in melanocytes of the dorsal skin in guinea pigs as an in vivo model.

## 2. Materials and Methods

### 2.1. Materials

**Figure 1:**
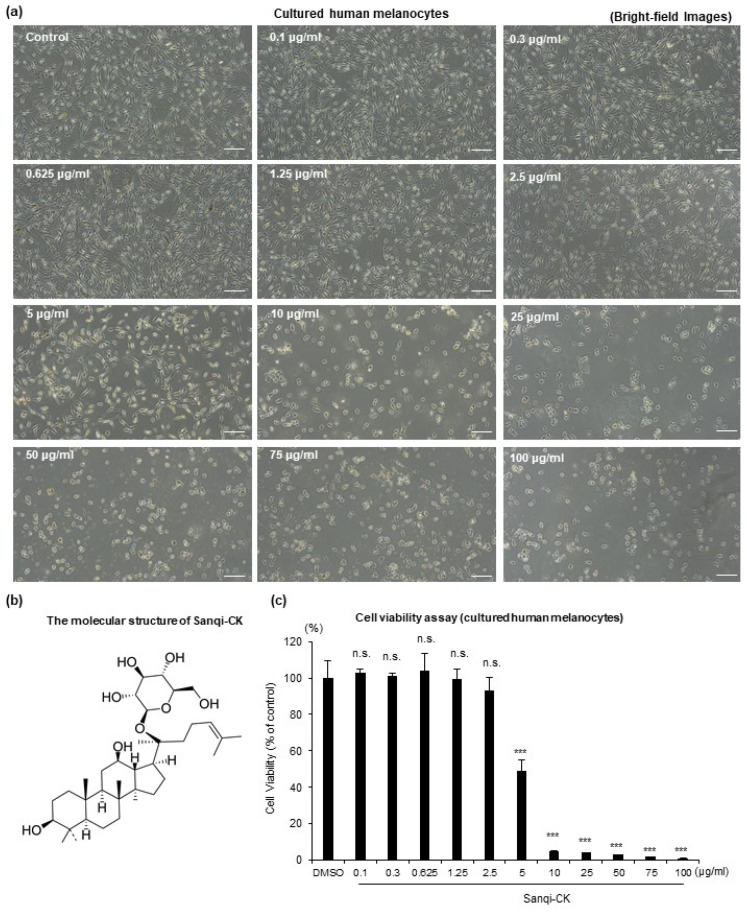
Cytotoxicity analyses by morphologic examination and MTT assay. Cultured human primary epidermal melanocytes (HEMn-MPs) were treated with increasing concentrations of Sanqi-CK (0.1–100 μg/mL) for 24 h. (**a**) Changes in melanocyte morphology were observed via bright-field microscopy; white bar, 50 μm. (**b**) Molecular structure of Sanqi-CK. (**c**) Cell viability was assessed by MTT assay. Data in (**c**) represent the results of three independent experiments and are shown as mean ± SD. n.s., no significant difference; *** *p* < 0.01; compared with control (DMSO) according to one-way analysis of variance (ANOVA) followed by Dunnett’s test.

Powdered CK (Figure 1b, purity: >98.0%) from Sanqi-extract (i.e., Sanqi-CK) was provided by Sichuan Weikeqi Biological Technology Co., Ltd. (Sichuan, China). It was dissolved in dimethyl sulfoxide (DMSO) for in vitro experiments and constituted into an ointment (2%) for in vivo experiments. For in vitro experiments, the final culture concentration of DMSO in all the treatments was 0.1% *v*/*v* in growth medium.

Rhododendrol (4-(4-hydroxyphenyl)-2-butanol, Rhododenol^®^) was kindly provided by Kanebo Cosmetics Inc. (Tokyo, Japan). A stock solution of 20% (*w*/*v*) rhododendrol was prepared in a 50% ethanol/50% sesame oil (*v*/*v*) mixture.

### 2.2. Cell Culture

Normal human primary skin epidermal melanocytes from moderately pigmented neonatal foreskin (HEMn-MPs) were purchased from Invitrogen (Thermo Fisher Scientific, Carlsbad, CA, USA) and cultured in Medium 254 (M-254-500; Thermo Fisher Scientific) supplemented with 1% (*v*/*v*) human melanocyte growth supplement (Thermo Fisher Scientific) at 37 °C in an atmosphere containing 5% (*v*/*v*) CO_2_. The melanocytes were used at passages 8 to 11. Cells were seeded into 6-well plates at a density of 5 × 10^5^ cells/well for 12 h before treatment. They were then treated with H_2_O_2_ (Wako, Osaka, Japan) and Sanqi-CK at indicated concentrations and for specific time periods prior to RNA and protein extraction.

### 2.3. MTT Assay

HEMn-MPs (1 × 10^4^ cells/well) were cultured in 96-well flat-bottom tissue culture plates. After completion of the experimental treatments, the cells were washed three times with cold phosphate-buffered solution (PBS), and cell viability was evaluated using the Cell Count Reagent SF colorimetric assay (Nacalai Tesque, Kyoto, Japan). Briefly, 10 μL of Cell Count Reagent SF was added to each well, and the cells were incubated for 2 h at 37 °C. Cell viability was determined colorimetrically by measuring OD_450_ with a microplate reader (Model 550; Bio-Rad Laboratories, Hercules, CA, USA). The percentage of viable cells was calculated as follows: percentage viable cells = T/C × 100, in which T and C were the mean OD_450_ of the treated and control groups, respectively.

### 2.4. Melanin Content Assay

To determine melanin content, cells were first dissolved in 200 μL of 1 N NaOH for 30 min at 100 °C to solubilize the melanin. Melanin content of the cell suspensions was then quantified by recording the absorbance at 405 nm, as described previously [21]. Melanin content was calculated and corrected based on the cell number.

### 2.5. Cell Counting Assay

Viable cell number was manually counted by trypan blue exclusion (Trypan Blue Dye, 0.40% solution, #1450013, Bio-Rad Laboratories, Hercules, CA, USA) using a disposable cell counting chamber (C-Chip, Neubauer Improved, DHC-N01N, NanoEn Tek Inc., Pleasanton, CA, USA).

### 2.6. L-Dopa Reaction

To evualuate the function of melanin synthesis in cultured melanocytes, after the indiated treatments, cells were incubated with 0.1% (*w*/*v*) levodopa (L-dopa) (Wako) for 1 h at 37 °C in an atmosphere containing 5% (*v*/*v*) CO_2_. The cells were then photographed under bright-field microscopy (Biozero 8000, Keyence Co., Osaka, Japan). 

### 2.7. Trosinase Activity Assay

The activity of tyrosinase in cultured melanocytes was measured using the Tyrosinase Activity Assay Kit (ab25899; Abcam, Tokyo, Japan) after the indicated treatments. Briefly, melanocytes (5 × 10^5^ cells) were homogenized with 500 μL of ice-cold Tyrosinase Assay buffer to perform lysis and kept on ice for 10 min, and the mixture was centrifuged at 10,000× *g* for 15 min at 4 °C. The tyrosinase activity in the supernatant was determined spectrophotometrically with absorbance at 510 nm using a Model 680 Microplate Reader (Bio-Rad Laboratories, Hercules, CA, USA), according to the manufacturer’s protocol. 

### 2.8. RNA Isolation and Real-Time RT-PCR Analysis

Total RNA from cell pellets was isolated using a Maxwell^®^ 16 LEV simplyRNA Tissue Kit (Promega, Madison, WI, USA), following the manufacturer’s instructions. RNA integrity was verified by gel electrophoresis. Total RNA (100 ng) was reverse-transcribed into first-strand cDNA (ReverTra Ace^®^ qPCR RT Master Mix; TOYOBO, Osaka, Japan). The primers used for real-time PCR were as follows: hTYR, sense 5′-TGACTCCAATTAGCCAGTTCCT-3′ and antisense 5′-GACAGCATTCCTTCTCCATCAG-3′; and hTYRP1, sense 5′-CTCAATGGCGAGTGGTCTGT-3′ and antisense 5′-TTCCAAGCACTGAGCGACAT-3′. Real-time PCR was conducted using a QuantStudio^®^5 Real-time PCR System (Applied Biosystems, CA, USA). Reactions were run in triplicate, during three independent experiments. The geometric mean of the housekeeping gene, GAPDH, was used as an internal control to normalize variability in expression levels.

### 2.9. Oxidative Stress Assessment

For cultured HEMn-MPs, oxidative stress was detected by live imaging using CellROX^®^ Green Reagent (#C10444; Thermo Fisher Scientific Inc.). Cells were treated with 5 µM CellROX^®^ Green Reagent for 30 min and then washed with PBS twice, treated with various concentrations of Sanqi-CK, and subsequently subjected to live-cell imaging by phase-contrast and confocal fluorescence microscopy (Biozero 8100; Keyence Co., Osaka, Japan). Hoechst 33342 (1:500 dilution; Invitrogen) was used to stain nuclei.

### 2.10. Measurement of Reduced and Oxidized Glutathione

Reduced glutathione (GSH) and oxidized glutathione (GSSG) levels in melanocytes were measured using the GSSG/GSH quantification kit (#G257; Dojindo Molecular Technologies Inc., Kumamoto, Japan) after the indicated treatments. Briefly, melanocytes (1 × 10^7^ cells) were homogenized with 80 μL of 10 mmol/L HCl and 20 μL of 5% (*v*/*v*) 5-sulfosalicylic acid, and the mixture was centrifuged at 8000× *g* for 10 min at 4 °C. Total glutathione and GSSG levels in the supernatant were determined spectrophotometrically using a Model 680 Microplate Reader (Bio-Rad Laboratories, Hercules, CA, USA), according to the manufacturer’s protocol. Concentrations of total glutathione and GSSG were determined using standard curves. GSH levels were calculated using the following formula: GSH (µmol/L) = total glutathione (µmol/L)−2 × GSSG (µmol/L). The GSH/GSSG ratio was then calculated.

### 2.11. Measurement of Glutathione Reductase Activity

Glutathione reductase (GR) activity in melanocytes was measured after the indicated treatments, using a Glutathione Reductase Assay Kit (ab83461; Abcam, Tokyo, Japan), according to the manufacturer’s instructions. Briefly, melanocytes (1 × 10^6^ cells) were homogenized with 150 μL of cold GR Assay Buffer, and the mixture was centrifuged at 10,000× *g* for 15 min at 4 °C. The supernatant was collected for assay. The absorbance was read at 405 nm in a Model 680 Microplate Reader (Bio-Rad Laboratories) to calculate the activity.

### 2.12. Animals and In Vivo Experimental Procedures

Three male 7-week-old JY-4 black guinea pigs were purchased from Tokyo Laboratory Animals Science (Tokyo, Japan) and maintained under specific pathogen-free conditions. In each guinea pig, four areas (each 2 cm × 2 cm) of the dorsal skin were used for four different experimental groups after clipping the hair with an electric shaver. Every experimental group contained three guinea pigs. The animals were treated topically with 20% (*w*/*v*) rhododendrol daily for 21 days to induce skin depigmentation and concomitantly given topical vehicle ointment or Sanqi-CK ointment (2%) daily for 21 days. Samples of skin were collected 24 h after the final treatment. Every effort was made to minimize animal suffering. The study was conducted according to the Guiding Principles for the Care and Use of Laboratory Animals and approved by the Committee for Animal Experiments at Osaka City University (Permit No. 18042, Osaka, Japan).

### 2.13. Measurement of Skin Color

Skin coloration in the treated area was quantified using a portable reflectance spectrophotometer (Konica CM-26d; Konica Minolta, Tokyo, Japan). The results are expressed as an L* value, which is a measure of skin lightness on a continuous black to white scale (0 = completely black; 100 = completely white).

### 2.14. Fontana-Masson Staining

Dorsal skin tissues were fixed in a 10% formalin neutral buffer solution (Wako Pure Chemicals, Osaka, Japan), embedded in paraffin, and sectioned on a microtome at a thickness of 5 μm. The sections were deparaffinized with xylene, rehydrated with a graded series of ethanol, and then subjected to Fontana-Masson staining using the Fontana-Masson Stain Kit (ab150669; Abcam), according to the manufacturer’s instructions.

### 2.15. Fluorescent Immunohistochemical Staining of Melanocytes

Dorsal skin tissues were fixed in 10% formaldehyde and embedded in paraffin, after which 5 μm sections were used for fluorescent immunohistochemical staining. Sections were incubated overnight at 4 °C with primary antibodies specific for tyrosine-related protein-1 (TYRP1) (HPA000937, 1:200 dilution; Sigma, St. Loius, MO, USA), and then incubated with the secondary antibody (anti-rabbit IgG Alex Fluor 488; Invitrogen, Thermo Fisher Scientific, Loughborough, United Kingdom) [21]. Sections were counterstained with Hoechst 33342 at a ratio of 1:500 (Invitrogen). The stained sections were visualized using a light microscope or a Biozero 8100 confocal microscope (Keyence Co., Osaka, Japan). 

### 2.16. Statistical Analyses

All experiments were repeated at least three times. Data are presented as mean ± SD. Statistical analyses were conducted using two-way analysis of variance to assess interactions between variables. Unpaired Student’s *t*-test (Microsoft Excel; Microsoft Corp., Redmond, WA, USA) was used to compare differences between two different groups. *p*-values < 0.05 were considered statistically significant.

## 3. Results

### 3.1. Sanqi-CK Attenuates H_2_O_2_-Induced Cytotoxicity in Human Primary Epidermal Melanocytes 

To investigate the effects of Sanqi-CK (Figure 1b) on the morphology of melanocytes, cultured normal human primary epidermal melanocytes (HEMn-MPs) were treated with various concentrations of Sanqi-CK (0.1–100 μg/mL) for 24 h, after which changes in morphology were observed via bright-field microscopy. No significant effects on melanocyte morphology were observed up to 2.5 μg/mL of Sanqi-CK; beyond this concentration, cytotoxicity was evident (Figure 1a). Furthermore, cell viability was determined using a 3-[4-dimethylthiazol-2-yl]-2,5-diphenyltetrazolium bromide (MTT) assay. Sanqi-CK up to 2.5 μg/mL did not affect viability, whereas viability decreased significantly above this concentration (Figure 1c). These results suggest that Sanqi-CK is nontoxic over a wide range of concentrations. A Sanqi-CK concentration of 1.25 μg/mL was chosen for subsequent experiments because this concentration demonstrated no effects on cell viability.

**Figure 2:**
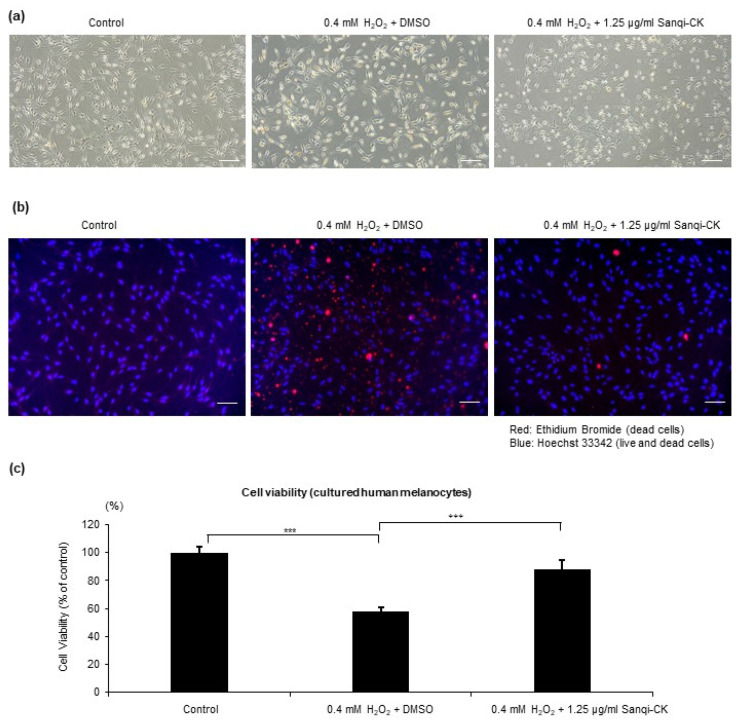
Protective effects of Sanqi-CK on cell survival of cultured human epidermal melanocytes (HEMn-MPs) against H_2_O_2_ treatment. The cells were pretreated with a nontoxic concentration of Sanqi-CK (1.25 μg/mL) for 6 h, followed by 0.4 mM H_2_O_2_ treatment for an additional 24 h. After these treatments, (**a**) melanocyte morphology was observed with bright-field microscopy; (**b**) cell staining was performed with ethidium bromide (red) and Hoechst 33342 (blue) and observed using confocal fluorescence microscopy; and (**c**) cell viability was evaluated by the MTT assay. White bar in (**a**,**b**), 50 μm. Data in (**c**) represent the results of three independent experiments and are shown as mean ± SD. *** *p* < 0.01; difference between the indicated two groups by Student’s *t*-test.

To investigate the effects of Sanqi-CK on the survival of melanocytes under oxidative stress, HEMn-MPs were treated with 1.25 µg/mL Sanqi-CK for 6 h and then exposed to 0.4 mM H_2_O_2_ for 24 h, after which morphology (Figure 2a,b) and viability (Figure 2c) of the cells were assessed. Morphologic observations with light microscopy showed that exposure of melanocytes to 0.4 mM H_2_O_2_ for 24 h resulted in membrane blebbing and cell shrinkage (Figure 2a, middle panel); these changes were rescued by pretreatment with 1.25 µg/mL Sanqi-CK (Figure 2a, right panel). Cell staining with ethidium bromide (for dead cells) and Hoechst 33342 (for both live and dead cells) showed that dead cells strikingly increased after 24 h treatment with 0.4 mM H_2_O_2_, whereas Sanqi-CK pretreatment reversed this H_2_O_2_-induced cell death (Figure 2b). The protective effects of Sanqi-CK against H_2_O_2_ were likewise confirmed using the MTT assay. The viability of HEMn-MPs decreased to 57.6% of control when subjected to 0.4 mM H_2_O_2_, but preincubation with Sanqi-CK for 6 h reduced these cytotoxic effects of 0.4 mM H_2_O_2_ (Figure 2c).

### 3.2. Sanqi-CK Attenuates H_2_O_2_-Induced Suppression of Melanogenesis in Human Primary Epidermal Melanocytes

**Figure 3:**
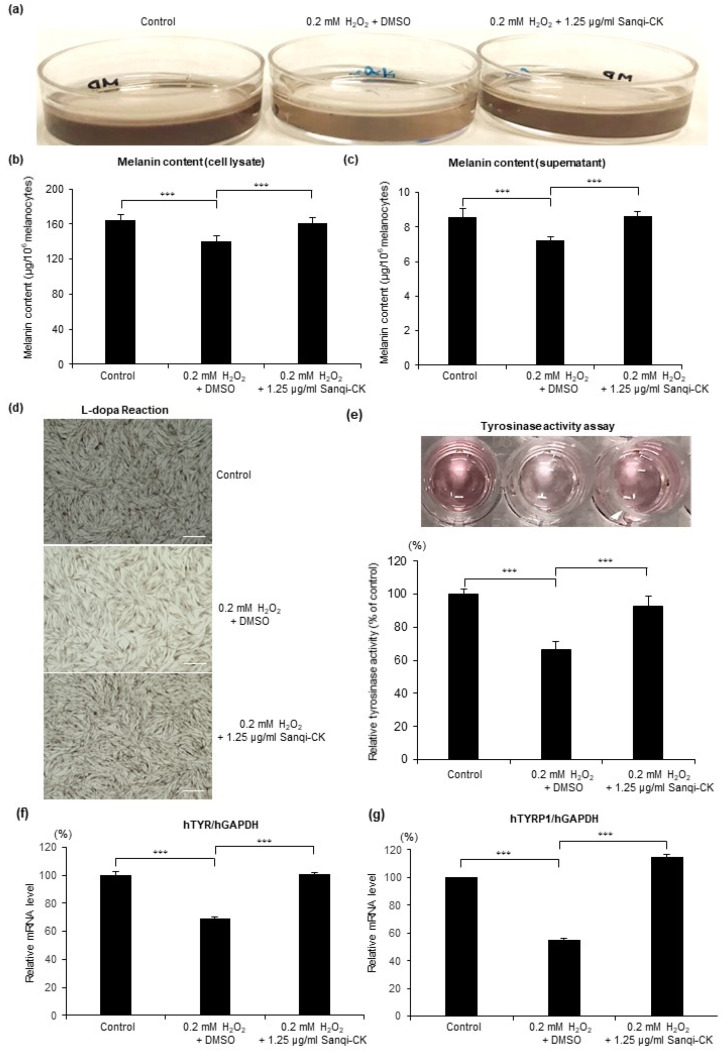
Protective effects of Sanqi-CK on melanin synthesis of cultured HEMn-MPs against H_2_O_2_ treatment. Melanocytes were pretreated with a nontoxic concentration of Sanqi-CK (1.25 μg/mL) for 6 h and then exposed to 0.2 mM H_2_O_2_ for 5 days. At the end of these treatments, (**a**) cultured melanocytes in medium were photographed; melanin content was quantified by melanin content assay in (**b**) cell lysates and (**c**) the culture medium; and (**d**) the L-dopa reaction was performed, with changes in pigmentation observed under bright-field microscopy. White bar in (**d**), 50 μm. At 24 h after exposure to the indicated treatments, tyrosinase activity (**e**) was evaluated by tyrosinase activity assay, the expression levels of key melanogenesis-related genes *TYR* (**f**) and *TYRP1* (**g**) were evaluated by real-time PCR analysis, with normalization to GAPDH expression. Data in (**b**,**c**,**e**–**g**) represent the results of three independent experiments and are shown as mean ± SD. *** *p* < 0.01; difference between the indicated two groups by Student’s *t*-test.

To investigate the effects of Sanqi-CK on the function of melanocytes under oxidative stress, the melanin content, L-dopa reaction, tyrosinase activity assay, and mRNA expression levels of key genes involved in melanogenesis were evaluated. HEMn-MPs were pretreated for 6 h with 1.25 µg/mL Sanqi-CK and then exposed to chronic oxidative stress by incubation with 0.2 mM H_2_O_2_ for 5 days. At the end of 5 days, cells exposed to 0.2 mM H_2_O_2_ were markedly less pigmented than control cells, and Sanqi-CK pretreatment markedly improved the culture medium color (Figure 3a). Melanin content was quantified using the melanin content assay of cell lysates (Figure 3b) and the culture medium supernatant (Figure 3c) of cultured HEMn-MPs. The results showed that chronic oxidative stress significantly decreased melanin content (Figure 3b,c), and Sanqi-CK pretreatment significantly attenuated H_2_O_2_-induced suppression of melanogenesis in both the culture medium and cell lysates (Figure 3b,c). 

The function of melanin synthesis was further evaluated by the darkness of cultured melanocytes after adding L-dopa as a substrate [22]. After 5-day exposure to 0.2 mM H_2_O_2_, the L-dopa reaction was performed (Figure 3d). Cells treated with 0.2 mM H_2_O_2_ exhibited markedly poor reactions to L-dopa, as they were less pigmented than control cells. Sanqi-CK pretreatment greatly improved the response to L-dopa (Figure 3d). Furthermore, after HEMn-MPs were pretreated for 6 h with Sanqi-CK and then exposed to 0.2 mM H_2_O_2_ for 24 h, the activity of the melanogenesis key enzyme tyrosinase was evaluated by tyrosinase activity assay (Figure 3e), and the expression levels of the key melanogenesis-related genes (*TYR* and *TYRP1*) were evaluated by real-time PCR analysis (Figure 3f,g). Tyrosinase activity and mRNA expression levels of both genes were significantly decreased in cells treated with 0.2 mM H_2_O_2_, compared with control cells, and Sanqi-CK pretreatment significantly inhibited H_2_O_2_-induced reduction of tyrosinase activity (Figure 3e) and H_2_O_2_-induced reduction of *TYR* and *TYRP1* expression (Figure 3f,g). These results therefore suggest that Sanqi-CK pretreatment can improve the dysfunction of melanocytes induced by chronic oxidative stress. 

### 3.3. Sanqi-CK Reduces H_2_O_2_-Induced Oxidative Stress in Human Primary Epidermal Melanocytes

**Figure 4:**
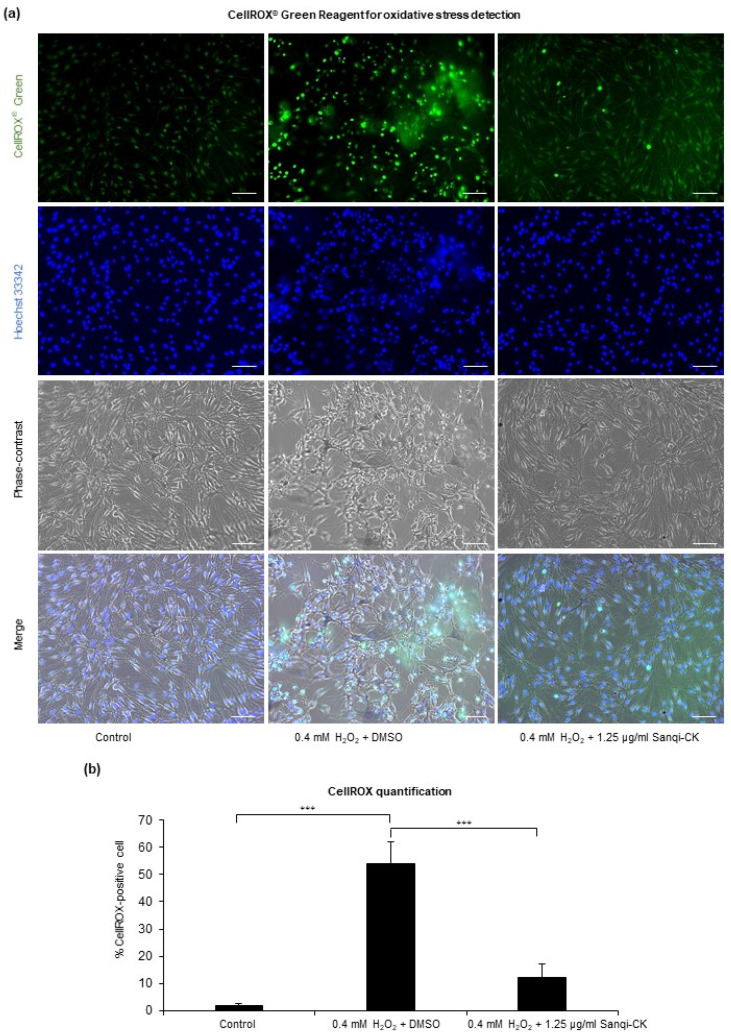
Effects of Sanqi-CK on oxidative stress in cultured human epidermal melanocytes (HEMn-MPs). The cells were pretreated with a nontoxic concentration of Sanqi-CK (1.25 μg/mL) for 6 h, followed by 0.4 mM H_2_O_2_ treatment for an additional 24 h. Oxidative stress in cultured cells was detected by CellROX^®^ Green reagent after exposure to the indicated treatments for 24 h. Nuclei were stained blue by Hoechst 33342. The cultured cells were photographed by phase-contrast and confocal fluorescence microscopy (**a**). Representative images obtained in three independent experiments are shown. White bar, 50 μm. Green signals indicate the presence of oxidative stress in cells. Percentage of CellROX-positive cells was quantified on 10 random fields per treatment group (**b**). All experiments were repeated at least three times and data presented in (**b**) are shown as mean ± SD. *** *p* < 0.01; difference between the indicated two groups by Student’s *t*-test.

Next, we examined whether Sanqi-CK protected melanocytes against oxidative stress. The effects of Sanqi-CK on oxidative stress were measured in cultured melanocytes treated with H_2_O_2_. CellROX^®^ Green Reagent was used to visualize intracellular oxidative stress in melanocytes by green fluorescence. As shown in Figure 4, exposing HEMn-MPs to 0.4 mM H_2_O_2_ for 24 h produced an obvious increase in green fluorescence. H_2_O_2_-induced green fluorescence was markedly attenuated in cells pretreated with 1.25 µg/mL Sanqi-CK for 6 h (Figure 4a,b), demonstrating the attenuating effects of Sanqi-CK on oxidative stress in melanocytes.

### 3.4. Sanqi-CK Modulates Redox Balance and Activates Glutathione Reductase in H_2_O_2_-Treated Human Primary Epidermal Melanocyte

**Figure 5:**
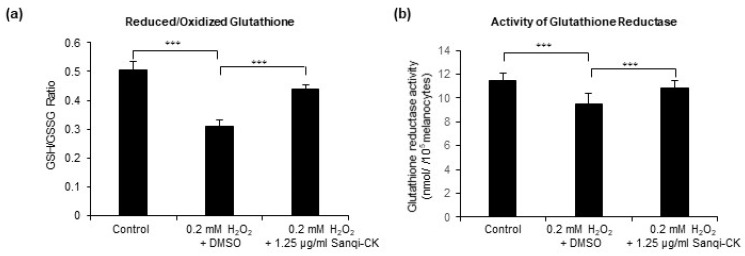
Effects of Sanqi-CK on redox status in cultured human epidermal melanocytes (HEMn-MPs). The cells were pretreated with a nontoxic concentration of Sanqi-CK (1.25 μg/mL) for 6 h, followed by 0.2 mM H_2_O_2_ treatment for an additional 24 h. After these treatments, we determined the (**a**) reduced glutathione/oxidized glutathione (GSH/GSSG) ratio and (**b**) glutathione reductase activity in the cells. Data in (**a**,**b**) represent the results of three independent experiments and are shown as mean ± SD. *** *p* < 0.01; difference between the indicated two groups by Student’s *t*-test.

To further explore underlying mechanisms of the effects of Sanqi-CK in reducing oxidative stress, we also assessed whether Sanqi-CK treatment modulates the major antioxidant defense mechanism in melanocytes. The glutathione redox cycle plays a key role in the reduction of intracellular hydroperoxides. Glutathione is one of the most widely studied antioxidants synthesized in the body and prevents oxidative stress by removing ROS. Glutathione is usually present in reduced form (GSH), but GSH is converted to its oxidized form (GSSG) during oxidative stress, and the GSH/GSSG ratio has been used as an indicator of oxidative stress [23]. Hence, we estimated the effects of Sanqi-CK on the GSH/GSSG ratio and observed a significant decrease in this ratio in H_2_O_2_-treated melanocytes (Figure 5a). In contrast, Sanqi-CK pretreatment prevented H_2_O_2_-mediated reduction of the GSH/GSSG ratio (Figure 5a), thereby indicating that Sanqi-CK maintained a well-regulated redox balance.

GR catalyzes the conversion of GSSG to GSH and is a pivotal enzyme in the cellular antioxidant defense mechanism. It plays a key role in response to oxidative stress by maintaining the intracellular pool of GSH [23]. We observed a significant decrease in GR activity in melanocytes after 24 h of H_2_O_2_ treatment (Figure 5b), which was rescued by Sanqi-CK pretreatment (Figure 5b).

### 3.5. Topical Sanqi-CK Ointment Treatment Suppresses Rhododendrol-Induced Depigmentation in Guinea Pigs

Rhododendrol was previously used as a skin-whitening cosmetic, but it was withdrawn from the market in 2013 because it causes a depigmentation disorder. A previous report suggested that the toxicity of rhododendrol towards melanocytes is the result of the production of cytotoxic ROS [24]. Moreover, topical rhododendrol has been used to develop animal models of leukoderma in keratin 14-promoter driven, stem cell factor transgenic (K14-SCF) mice [25,26] and guinea pigs [27].

**Figure 6:**
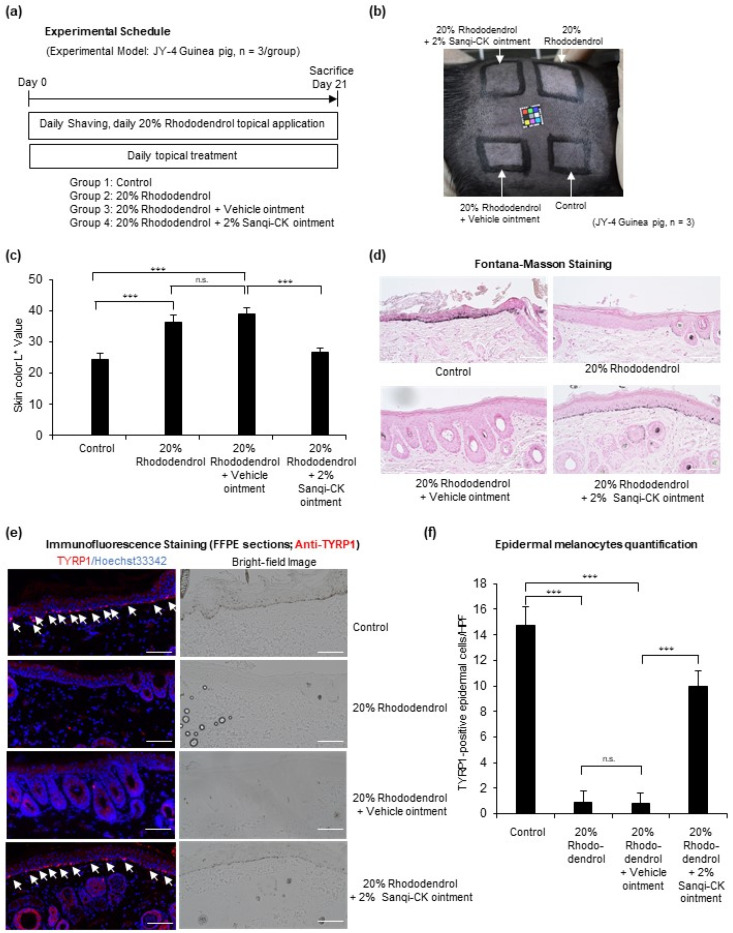
Effects of Sanqi-CK ointment in a rhododendrol-induced leukoderma guinea pig model. (**a**) Experimental schedule; (**b**) macroscopic appearance of skin color; (**c**) L* value of skin color in the indicated treatment groups; (**d**) Fontana-Masson staining of skin sections in the indicated treatment groups, with melanin appearing black; and (**e**) melanocytes stained with anti-TYRP1 antibody (in red) and nuclei stained with Hoechst 33342 (in blue), with bright-field images shown in the lower panel. White arrows indicate TYRP1-positive epidermal melanocytes. The red signal outside the skin is from keratin autofluorescence. Representative images of three independent experiments performed are shown in (**b**,**d**,**e**). White bar in (**d**,**e**), 100 μm; (**f**) TYRP1-positive epidermal melanocytes were counted in 10 non-contiguous random grids under high-power magnification fields (HPF, 200×) by confocal microscopy. The number of epidermal melanocytes per 10 hyper-power microscopic fields is shown in the histogram. Data in (**c**,**f**) are shown as mean ± SD. n.s., no significant difference; *** *p* < 0.01; difference between the indicated two groups by Student’s *t*-test.

To confirm the protective effects of Sanqi-CK in vivo, we investigated whether topical Sanqi-CK treatment could protect melanocytes against rhododendrol-induced cytotoxicity and suppress rhododendrol-induced leukoderma development in black guinea pigs (Strain: JY-4) (Figure 6a). After daily topical application of 20% rhododendrol for 21 days, obvious skin depigmentation was observed; however, concomitant administration of 2% Sanqi-CK ointment markedly suppressed this rhododendrol-induced depigmentation (Figure 6b). In the areas where vehicle ointment was applied (instead of Sanqi-CK), topical 20% rhododendrol induced a significant increase in skin lightness, as determined by the skin color L* value (Figure 6c). However, the rhododendrol-induced increase in skin lightness was significantly suppressed in areas treated with topical Sanqi-CK (Figure 6c). Further, Fontana-Masson staining for melanin showed that topical Sanqi-CK treatment markedly attenuated rhododendrol-induced decreases in melanin content in rhododendrol-applied skin (Figure 6d). Histochemical immunostaining of epidermal melanocytes with anti-TYRP1 antibody, a standard marker for melanocytes (Figure 6e), revealed complete disappearance of melanocytes after 21 days of rhododendrol topical application. In contrast, topical Sanqi-CK treatment rescued the disappearance of melanocytes in the epidermis of guinea pig skin (Figure 6e,f).

## 4. Discussion

This study was the first to demonstrate that Sanqi-CK can protect human melanocytes from H_2_O_2_-induced cell damage by modulating redox balance between GSH and GSSG and by activating GR activity. In addition, topical treatment with 2% Sanqi-CK ointment markedly suppressed rhododendrol-induced skin depigmentation in guinea pigs. Oxidative stress has been demonstrated to play a major role in melanocyte damage, which leads to loss of functional melanocytes in vitiligo [7]. Therefore, antioxidant-based therapy has evolved as a promising strategy for treating vitiligo, and the search for new agents capable of reducing oxidative stress in melanocytes has become an important research focus.

Plant products are generally considered to be less toxic and less prone to side effects than drugs manufactured by chemical synthesis. The potential therapeutic and preventive benefits of plant-based medications have been the subject of an extensive number of studies, and many natural constituents with significant pharmacologic activity have been uncovered [28]. Sanqi, also known as radix notoginseng (the roots of *Panax notoginseng*), is a widely used traditional Chinese medicine. This extract from the roots of the herb Sanqi has been used as to reduce blood stasis, decrease bleeding, reduce swelling, and alleviate pain in China for thousands of years [29,30]. In addition, it has been used extensively in medical research or clinical settings for a number of disorders, including hypertension, atherosclerosis, diabetes, acute lung injury, cancer, and cardiovascular diseases. Injections and capsules of Sanqi extract have become commercially available and are widely used in clinical practice [30,31]. This agent attracted our attention because Sanqi-CK has been recently reported to be beneficial in various oxidative-stress-related conditions. For instance, it was shown to alleviate hypolipidemia-associated oxidative stress in high-fat-fed rats through improving hepatic antioxidant activity via increasing superoxide dismutase levels and glutathione peroxidase activities [18]; protect against nephropathy through significantly reducing oxidative stress [19]; and enhance memory through activating the antioxidant system [20]. However, no previous report examined whether Sanqi-CK can protect melanocytes from oxidative stress. Thus, the purpose of the present study was to evaluate the effects of Sanqi-CK on melanocytes under oxidative stress.

In the present study, H_2_O_2_ was used to induce oxidative stress in cultured human primary melanocytes in vitro, and topical rhododendrol application was used to induce oxidative stress in epidermal melanocytes in vivo. We found that after 24 h incubation with 0.4 mM H_2_O_2_, cultured normal epidermal human melanocytes displayed a marked increase in oxidative stress (detected by CellROX^®^ staining) and dramatic cytotoxicity. In addition, melanin synthesis was strikingly inhibited after 5 days of 0.2 mM H_2_O_2_ stimulation in these cultured melanocytes. However, treatment with a nontoxic concentration of Sanqi-CK (1.25 µg/mL) clearly attenuated the H_2_O_2_-induced oxidative stress, cytotoxicity, and reduced melanin production. These data therefore suggest that Sanqi-CK pretreatment can reduce the accumulation of oxidative stress and alleviate oxidative-stress-induced loss of function (melanin production) in melanocytes. 

We also developed a topical Sanqi-CK ointment and examined the effects of this ointment on skin and melanocytes under oxidative stress. We used a guinea pig model because epidermal melanocytes do not reside in the dorsal skin of mice; black guinea pigs (strain, JY-4) have epidermal melanocytes in their entire body skin. A cosmetic lotion containing rhododendrol was used to produce rhododendrol-induced leukoderma, a depigmentation disorder caused by increased oxidative stress and cell cytotoxicity in melanocytes. Rhododendrol toxicity in melanocytes is known to be prevented by antioxidants [24]. We previously reported the development of this topical-rhododendrol-induced depigmentation animal model in guinea pigs [27], which is recognized as a good animal model for screening antioxidant agents able to protect melanocytes. Twenty-one days of topical 20% rhododendrol application induced obvious depigmentation, marked reduction in the number of epidermal melanocytes, and marked reduction in melanin pigment in the epidermis. However, topical 2% Sanqi-CK ointment attenuated rhododendrol-induced melanocyte cell death and skin depigmentation. These results strongly suggest that Sanqi-CK protects against oxidative-stress-induced cytotoxicity and has antioxidant and anti-cytotoxic effects in melanocytes.

Regarding the mechanism of the protective effects of Sanqi-CK on melanocytes, we found that GR activity and the GSH/GSSG ratio were reduced in H_2_O_2_-treated melanocytes; however, addition of Sanqi-CK (1.25 µg/mL) significantly reversed these H_2_O_2_-induced effects. GR is an enzyme that catalyzes the reduction of GSSG to GSH [23]. GSH is a critical molecule for resisting oxidative stress and maintaining the reducing environment of a cell [32]. Thus, GR is an important antioxidative enzyme and plays an integral role in maintaining the appropriate redox status in cells. Glutathione levels and reduced/oxidized glutathione balance status were previously reported to be abnormal in vitiligo [33] and rhododendrol-induced depigmentation [34]. Our results therefore suggest that Sanqi-CK can protect melanocytes against oxidative stress by enhancing glutathione reduction and modulating the redox status, which may be a good preventive or treatment strategy for vitiligo and rhododendrol-induced leukoderma.

## Data Availability

The data presented in this study are available on request from the corresponding author.
